# Regulated Intramembrane Proteolysis of ACE2: A Potential Mechanism Contributing to COVID-19 Pathogenesis?

**DOI:** 10.3389/fimmu.2021.612807

**Published:** 2021-06-07

**Authors:** Sandra M. Gonzalez, Abu Bakar Siddik, Ruey-Chyi Su

**Affiliations:** ^1^ Department of Medical Microbiology and Infectious Diseases, University of Manitobag, Winnipe, MB, Canada; ^2^ National HIV and Retrovirology Laboratories, J.C. Wilt Infectious Diseases Research Centre, National Microbiology Laboratories, Public Health Agency of Canada, Winnipeg, MB, Canada

**Keywords:** regulated intramembrane proteolysis, ACE2, SARS-CoV-2, COVID-19, ectodomain shedding, endodomain cleavage

## Abstract

Since being identified as a key receptor for SARS-CoV-2, Angiotensin converting enzyme 2 (ACE2) has been studied as one of the potential targets for the development of preventative and/or treatment options. Tissue expression of ACE2 and the amino acids interacting with the spike protein of SARS-CoV-2 have been mapped. Furthermore, the recombinant soluble extracellular domain of ACE2 is already in phase 2 trials as a treatment for SARS-CoV-2 infection. Most studies have continued to focus on the ACE2 extracellular domain, which is known to play key roles in the renin angiotensin system and in amino acid uptake. However, few also found ACE2 to have an immune-modulatory function and its intracellular tail may be one of the signaling molecules in regulating cellular activation. The implication of its immune-modulatory role in preventing the cytokine-storm, observed in severe COVID-19 disease outcomes requires further investigation. This review focuses on the regulated proteolytic cleavage of ACE2 upon binding to inducer(s), such as the spike protein of SARS-CoV, the potential of cleaved ACE2 intracellular subdomain in regulating cellular function, and the ACE2’s immune-modulatory function. This knowledge is critical for targeting ACE2 levels for developing prophylactic treatment or preventative measures in SARS-CoV infections.

## Introduction

The recent pandemic caused by the Severe Acute Respiratory Syndrome Coronavirus-2 (SARS-CoV-2) has become a catastrophic event threating global health, reaching millions of infected individuals worldwide with a variable temporal estimates of case-fatality rate among the affected countries oscillating between 1.6% and 31.4% (1, 2) and requiring the establishment of repeated lockdowns in many countries to control the spreading and to reduce the impact of the infection. Currently, many countries overcame a second wave of infections and there is a potential risk for more waves to come as several recent described variants of the virus have been reported in different countries (3, 4). Nonetheless, the exceptional efficacy exhibited by the available vaccines in reducing severity of coronavirus disease-19 (COVID-19), and deaths has brought hope on overcoming this pandemic in a shorter period of time (5–7). Challenges for global distribution and access to vaccines remain and it is imperative to vaccinate as many people as possible to avoid emergence of new variants and surpass the pandemic.

Our knowledge of SARS-CoV-2 remains limited. However, lessons learned from previous coronavirus (CoV) outbreaks, mainly SARS-CoV, which caused the outbreak in 2002-2003 and shares close genotypic and phenotypic similarities with SARS-CoV-2 (8) can inform the immunopathogenic mechanisms triggered during SARS-CoV-2 infection and the factors potentially responsible for its rapid transmission and the severe COVID-19 outcomes. This knowledge is crucial for the development of preventive strategies and prophylactic treatment options.

Like SARS-CoV, SARS-CoV-2 uses the angiotensin-converting-enzyme 2 (ACE2) on the target cell as a binding and entry receptor. Both interact with ACE2 *via* the receptor-binding domain (RBD) of the viral spike (S) protein, specifically, the subunit 1 (S1) (9). Following this initial interaction, virions are internalized in the acidic endocytic compartments where proteolytic cleavage of the S protein into S1 and S2 subunits by cellular transmembrane protease serine 2 (TMPRSS2) occurs. Cleavage of S protein allows the exposure of the fusion domain of the S2 subunit and the subsequent fusion of the viral envelope with the cellular membrane. This process is similar for both SARS-CoV and SARS-CoV-2 (9), and is crucial for the release of viral ssRNA genome into cytosol (10). Interestingly, preventing the S protein cleavage by inhibiting the TMPRSS2 and the cysteine proteases CATHEPSIN B/L activity *in vitro* can block the SARS-CoV from entering the cells (11) but cannot completely block SARS-CoV-2 (9), suggesting that SARS-CoV-2 has other cleavage site(s). In support, proteomic analyses identified potential FURIN cleavage sites in SARS-CoV-2 S protein (12) and FURIN protease was shown to cleave MERS-CoV S protein (13). Although inhibitor of FURIN activity could reduce SARS-CoV-2 replication *in vitro*, it remains to be determined if SARS-CoV-2 infection also utilizes FURIN or other proteases (14, 15).

Furthermore, other molecules such as the transmembrane receptor CD147 (basigin) expressed by epithelial and immune cells from lung and skin, and the glycoprotein CD26, highly expressed by CD4 and CD8 T cells and innate lymphoid cells (ILC) (16), have been suggested to facilitate SARS-CoV-2 entry *via* interacting with the viral S protein (17, 18). Indeed, the antibody against CD147 inhibited *in vitro* SARS-CoV-2 infection (17). Nonetheless, the use of these alternative receptors by SARS-CoV-2 requires further exploration and confirmation in patients with COVID-19.

The proteomic activity of TMPRSS2 and the cleavage of ACE2 facilitate the internalization of SARS-CoV Sp S1 subunit bound to ACE2 (19). Cleavage-resistant ACE2 mutant failed to facilitate the internalization of the Sp S1 subunit, but the protease that cleaves ACE2 during SARS-CoV-2 infection remains debatable (19, 20). Together, this *in vitro* data suggest that during SARS-CoV infection, ACE2 cleavage occurs following Sp S1 binding and is followed by the internalization of Sp S1. SARS-CoV-2 Sp binds more strongly to ACE2 than does SARS-CoV Sp (21, 22); it remains to be sought if internalization of SARS-CoV-2 also requires the proteolytic activity of TMPRSS2 and/or the cleavage of ACE2. Curiously, the internalization of SARS-CoV–1-ACE2 was shown to up-regulate the activity of a disintegrin and metallopeptidase 17 (ADAM17) (20), resulting in increased proteolysis of the ACE2 ectodomain (19, 23, 24) and decreased surface ACE2. Reduced cellular ACE2 level is associated with heightened renin-angiotensin system (23) due to increased tissue and serum level of Ang II (25, 26), which is associated with inflammation and tissue damage (27–29).

**Figure 1:**
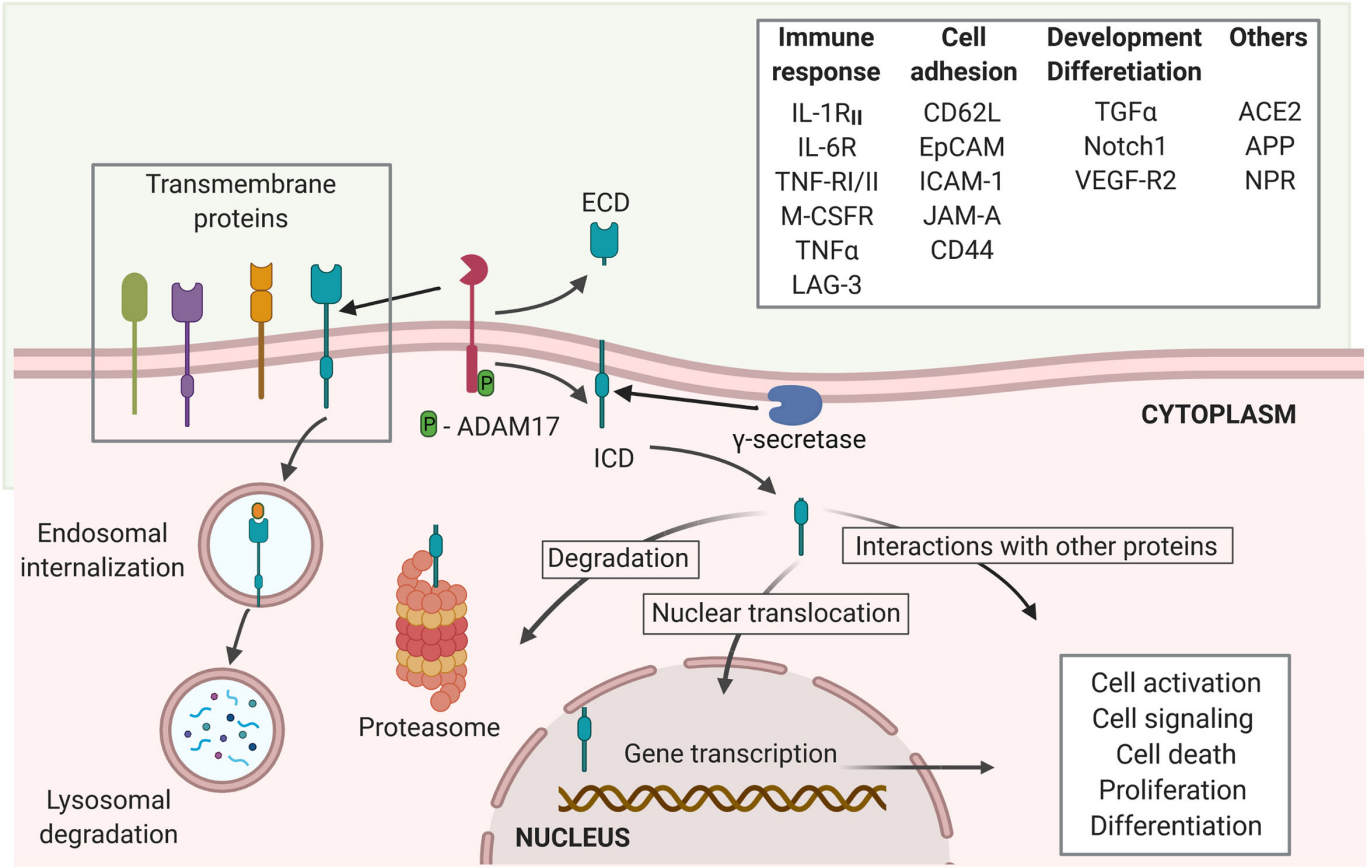
Regulated intramembrane proteolysis process and proteins cleaved by ADAM17. Several proteins are targets of ADAM17 cleavage, including proteins involved in immune responses, cell adhesion and cellular development and differentiation, among others. The proteolytic activity of ADAM17 can be triggered through its phosphorylation by PKL2, PKC, and MAPKs. Activated ADAM17 cleaves several transmembrane proteins leading to the release of an extracellular domain (ECD) and the membrane retention of an intracellular cytoplasmic domain (ICD). The γ-secretase multi-protein complex removes this domain from the membrane allowing it to migrate to the cytoplasm where it can either be degraded at the proteasome or interact with other cytoplasmic proteins. Moreover, the ICD can trans-locate into the nuclei to induce transcriptional expression of several genes related to cell activation, signaling, death, proliferation and differentiation (created with BioRender.com).

ACE2 is a potential target of the regulated intramembrane proteolysis (RIP). RIP is a signaling mechanism that follows the shedding of the extracellular domain of several trans-membrane proteins, such as the Interleukin-1 receptor type II (IL-1R_II_), the IL-6R, TNFα and its receptors TNF-RI and TNF-RII, the Amyloid precursor protein (APP), the Epithelial adhesion molecule (EpCAM), Notch 1, among many others (30, 31). The cleavage of the extracellular domain of the target proteins of RIP to release the ectodomain is mostly mediated by proteases of ADAM family, like ADAM17, matrix metalloproteases, and the aspartyl proteases β-site APP-cleaving enzymes (BACE) (24, 30). The second cleavage is the proteolysis of the intracellular domain, resulting in the release of an intracellular short carboxy-terminal fragment removed from the intra-membrane subdomain (30). It is mainly mediated by γ-secretase multiprotein complex (24). While the released extracellular domains can induce activation and signaling of cells binding to these fragments, the intracellular subdomains can either be degraded at the proteasome or lysosomes or induce cytoplasmic signaling through interaction with other molecules. Some released intracellular subdomains of proteins that undergo RIP have been shown to translocate into the nuclei to act as messengers and elicit biologic responses, such as changes in cellular transcriptional profile and signaling, and thus, influence the cell fate (24, 30, 31) (**Figure 1**). The outcome of the endodomain may depend on the specific cleaved protein and regulatory signals, which remain a gap of knowledge in the RIP research. Several studies recognize that ADAM17, also known as tumor necrosis factor α-converting enzyme (TACE) is the main protease, responsible for the cleavage of ACE2 (19, 20, 32, 33), even though TMPRSS2, the type II membrane serine protease can also cleave ACE2 (19, 33). The conformational features of ACE2 and its cleavage mediated by ADAM17 (19) suggest that ACE2 may be a target of RIP (30). To support ACE2 being a target of RIP, evidence should be obtained that following the shedding of ectodomain, the truncated ACE2 is also targeted for intramembrane proteolysis, releasing a soluble ACE2 C-terminal fragment. However, whether the intracellular (endo-) domain of ACE2 is cleaved following the binding of SARS-CoV-2 Sp and if the ACE2 endo-domain has a regulatory role, like other targets of RIP remains to be sought.

This review focuses on the proteolysis of ACE2 in physiological and disease contexts and summarizes findings that support the RIP of ACE2 during SARS-CoV-2 and other CoVs infections. Moreover, we propose here a hypothesis of the potential effects of RIP of ACE2 in cellular fate and COVID-19 disease progression and severity.

## ACE2 Cleavage and the Enzymes Involved

ACE2 is a zinc metallopeptidase, type I transmembrane protein widely expressed in several tissues and organs, such as vascular endothelia and cardiovascular tissue, brain, the epithelial cells from oral and nasopharyngeal mucosa, lungs, kidney, gastrointestinal mucosa, Langerhans of the pancreatic tissue, and bone, among others (32, 34–38). There is no significant expression of ACE2 in the lymphoid organs and hematopoietic cells but macrophages (36, 37). Human *ACE2* is a 40 kb gene, mapped to chromsomeXp22. *ACE2* transcripts are found in 72 human tissues (39). It encodes 22 introns and 18 exons (40). ACE2 protein consists of ~805 amino acids encoding a large extracellular domain (also termed ectodomain) (723aa), a transmembrane domain (21aa) and a short cytoplasmic domain (also termed endodomain) (44aa) (40). Both ACE2 and its homolog, ACE are fundamental regulators of the renin-angiotensin system (RAS) that controls blood pressure homeostasis (26, 41) and the main function of ACE2/angiotensin (1-7) [Ang(1-7)] axis is to counter-balance the effects of ACE/Ang II axis (42). Both enzymes differ in their substrate specificity: ACE converts Ang I to the potent vasoconstrictor, Ang II; ACE2 then cleaves Ang II to generate the vasodilator, Ang(1-7) (27, 28, 41). ACE2 has also been shown to cleave Ang I directly, bypassing ACE function to generate Ang(1-9), but at a much-reduced efficiency compared to the cleavage of Ang II (43). While inhibition of ACE results in protective cardiovascular effects including reduced blood pressure and lower levels of Ang II, reduced ACE2 expression is reported in people with hypertension. However, deletion of *Ace2* expression in mouse model did not directly cause hypertension but caused enhanced susceptibility to Ang II-induced hypertension (44). Moreover, reduced ACE2 expression is associated with cardiac dysfunction and heart failure that could indirectly result in hypertension (42, 45, 46). These support the contrasting effects of ACE2/Ang (1-7) and ACE/Ang II axes (42, 45, 46). Moreover, ACE2 cleaves several other bioactive and physiologically important peptides, such as bradykinin, an endothelium-dependent vasodilator, the hypotensive peptide apelin-13, the opioid peptides dynorphin A and β-casomorphin, the neuropeptide, neurotensin, and the amyloid-β peptide involved in Alzheimer disease (43, 47). As mentioned above, ACE2 has been exploited by coronavirus, such as SARS-CoV, SARS-CoV-2 and HNL-63-CoV, which causes common cold, for viral entry (9, 48). Hence, a better understanding of ACE2 signaling and function is essential for preventative strategies involving the modulation of ACE2 levels, binding and signaling.

An interesting feature shared between ACE2 and ACE is the shedding of their extracellular domain. ACE is cleaved by the ACE secretase (43), a sheddase related but different from ADAM17, which is the main protease for ACE2 cleavage (25). ADAM17 is not shown to be involved in the shedding of ACE (49), partially explained by the minimal sequence homology between ACE and ACE2 at the cleavage sites (43). Both exhibit only ~42% amino acid homology at their extracellular catalytic domain (40). Whether ACE secretase could induce ACE2 shedding is currently unknown but unlikely (43).

In addition to ADAM17, TMPRSS2, human airway trypsin (HAT) and hepsin have also been shown to cleave ACE2. TMPRSS2 can interfere with ADAM17-mediated cleavage of ACE2 and has been shown to be exploited by CoVs to cleave the S protein, required for viral entry into host cells (19, 33). All these three enzymes, TMPRSS2, HAT and hepsin cleave ACE2 at the same catalytic site, which differs from the proteolytic site for ADAM17. Thus, differential outcomes of such cleavages compared to ADAM17-mediated shedding may occur (19). It is not clear whether these 3 enzymes have redundant roles in the ACE2 signaling and whether HAT and hepsin could also cleave S protein to facilitate CoV entry. The role of ADAM17 in facilitating SARS-CoV-2 infection was examined in a recent study using inhibitor (GW280264X) and the siRNA against ADAM17 to block SARS-CoV-2 infection of HK-2, a human kidney cell line, by >90% (50). Data presented in this study suggests that ADAM17 is the primary sheddase for ACE2.

The consequential events triggered by ACE2 cleavage remain poorly understood, so do the effects of shedding extracellular and the intracellular ACE2 domains. Since similar shedding processes have been described for other membrane proteins, they could potentially provide some insights on the destiny of ACE2 fragments and their functions.

## The Physiological Role of ACE2 ECTODOMAIN and the Potential Implications of ACE2 ENDODOMAIN

Following the cleavage of the extracellular domains from an ever-increasing number of proteins, RIP processing can lead to cellular signaling and modulation of the surrounding microenvironment (30). RIP is a crucial event in immune response, cellular development and differentiation, and cell adhesion, as well as in several diseases including cancer, Alzheimer and others (24, 30, 51, 52). Although it has not been completely established if ACE2 shedding corresponds to a RIP event, cleavage of several proteins by ADAM17 is accompanied by an intramembrane proteolysis mediated by the multiprotein γ-secretase complex (24) (**Figure 1**). In a mouse model of neurogenic hypertension, deoxycorticosterone acetate-salt treatment caused significant increases in ADAM17 expression and activity in the hypothalamus and decreased hypothalamic ACE2 activity and expression, which are accompanied by increases in blood pressure, hypothalamic Ang II levels, and inflammation, and impaired autonomic dysfunction (53). Reduced ACE2 expression and activity in the brain also occurred in parallel with an increase of shed ACE2 ectodomains and enzymatic activity in the cerebrospinal fluid (53). Chronic knockdown of ADAM17 in the brain was able to blunt the development of hypertension and restored ACE2 activity and baroreflex function (53). The caveat with chronic knockdown of ADAM17 is that there are numerous substrates for ADAM17 and hence, ADAM17 affects many systems, including cytokines that may play a role in hypertension. However, similar effects in blunting the development of hypertension and restoring baroreflex function were also observed when *Ace2* was overexpressed in the neurons (53). Nonetheless, in this model, it is not clear if the pathogenesis was caused by reduced ACE2 and Ang (1-7) expression and activity in the brain or the increase of Ang II and/or ACE2 ectodomains in the cerebrospinal fluids. The recombinant ACE2 (rhACE2) will be a great tool to address this question (54). Moreover, the effect of other inflammatory pathways that are also modulated by ADAM17 activity on brain inflammation, cannot be ruled out in this model (24, 51, 53).

Spontaneous, constitutive cleavage of ACE2 ectodomain can occur in the absence of disease and may be tissue specific. Soluble ACE2 ectodomain with enzymatic activities are detected in human bronchoalveolar lavage and urine samples from healthy donors (55, 56). In the culture of mouse proximal tubular cells, the low level, constitutive cleavage of ACE2 ectodomain was detected (57, 58) but could be further augmented by high levels of D-glucose or Ang II (58). Adding high concentrations of Ang II (10^−7^ M) or D-glucose (25mM) to the culture of mouse proximal tubular cells resulted in increased ADAM17 activity in the cell lysate and augmented release of ACE2 extracellular subdomains and ACE2 enzymatic activity in the culture media in a time-dependent manner (58). Hyperactivity of Ang II signaling pathways have been shown in both clinical trials and animal models of diabetes to contribute to the development of diabetes and diabetic complications (59). In addition, increased soluble ACE2 is found in the urine of patients with diabetes and/or renal diseases (59–63). Although these observations imply a role for ACE2 shedding in diabetes, whether ACE2 cleavage has a compensatory or a pathogenic role in the development of diabetes remain to be sought.

In addition to high glucose-D and Ang II, phorbol ester, ionomycin, endotoxin and IL-1β & TNF-α could also acutely increase the release of ACE2 ectodomain from primary, polarized human airway epithelial cells and Calu-3 (human lung adenocarcinoma cell line) (55). Cleaved ACE2 ectodomain was detected in the apical secretions of polarized epithelial cells in culture and in the human bronchoalveolar lavage. Inhibition assay using DPC333 (1.5 nM) and GI254023 (5μM), inhibitors for ADAM17 and ADAM10, respectively (55), showed that ADAM17 activity is required in both the constitutive and acutely induced ACE2 shedding but inhibition of either ADAM17 or ADAM10 prevented the release of ACE2 ectodomain induced by PMA or ionomycin, respectively (24, 52). Evidence supporting ADAM17’s role in regulated proteolysis of ACE2 is strengthened by the observations that siRNA specific for ADAM17 reduced PMA-induced ACE2 shedding and overexpression of ADAM17 in HEK293 cells increased ACE2 shedding (24). On the other hand, inhibition or siRNA-mediate knockdown of ADAM10 expression had no impact on PMA-triggered shedding of ACE2 (24, 55, 63). These studies are in agreement that while ADAM17 may be the main sheddase for cleaving ACE2 ectodomain constitutively at low level or in response to stimulus, such as PMA, ADAM10 is involved in ACE2 shedding in response to ionomycin (24, 55, 63). ADAM10 is also involved in ionomycin-induced shedding of CX3CL1 and CXCL16 from leukocytes and EGFR-ligand shedding from fibroblasts (64, 65). The response to PMA and ionomycin stimulation of regulated proteolysis of ACE2 ectodomain further suggests the involvement of proteinase kinase C pathway and intracellular calcium signaling in regulating ACE2 shedding. The shedding of ACE2 ectodomain from mice cardiomyocytes could also be induced by Ang II, which was shown to increase the phosphorylation and activity of ADAM17 (25, 66). In patients with heart failure, increased ACE2 ectodomain was reported in plasma, with increased ACE2 enzymatic activity, suggesting that excessive shedding and extracellular release of the ACE2 ectodomain is associated with diseases (25, 62). It remains to be shown whether the enzymatic activity of circulatory ACE2 ectodomains has any physiological or pathological significance. It is possible that shedding is one of the mechanisms for regulating ACE2 activity on the cell surface (67). Paradoxically, while surface ACE2 facilitates SARS-CoV entry, soluble ACE2 ectodomain has been shown to block SARS-CoV infection of cells. In contrast, a recent study showed that soluble ACE2 facilitate SARS-CoV-2 uptake *via* binding to Ang II type 1 receptor (AT1R) (50). Recombinant human ACE2 has been tested in a phase 2-3 trial in acute respiratory distress syndrome with interesting results (68), and a pilot trial has been launched in COVID-19 (NCT04287686) (69, 70).

Like other proteins that undergo RIP, there is some evidence suggesting that following the release of ACE2 ectodomain, the ACE2 endodomain is also cleaved off the transmembrane subdomain and released into cytosol (19). The γ-secretase complex, a multi-subunit protease complex that catalyzes the proteolysis of single-pass transmembrane proteins at residues within the transmembrane domain, catalyzes the RIP of several proteins, such as E-Cadherin, EpCAM, amyloid precursor protein (APP) and CD44 (52, 71). The endodomain of these proteins have been shown to facilitate nuclear signaling in altering cellular gene expression under specific cellular conditions (51). The release of ACE2 endodomain needs to be confirmed, and the fate of ACE2 endodomain remains to be sought. Our current knowledge of how the activity of γ-secretase complex is triggered to mediate intramembrane proteolysis is limited; the molecular mechanism(s) leading up to the cleavage of ACE2 endodomain are yet to be defined (**Figure 1**). The intracellular ACE2 endodomains were also detected following the TMPRSS2 mediated cleavage of ACE2 ectodomain in 293 T cells transfected with full-length ACE2 (19). Furthermore, ADAM17 and TMPRSS2 do not seem to share the same cleavage site on the ACE2 ectodomain (19). Interestingly, Heurich et al. reported the presence of an ACE2 C-terminal fragment (13kDa) in the cellular lysates following TMPRSS2-mediated ACE2 cleavage, and this ACE2 C-terminal fragment was absent in the cell lysate following ADAM17-mediated ACE2 cleavage (19). It suggests that the trigger for the shedding of the ACE2 ectodomain may affect the fate of ACE2 intracellular domain. This is further supported by evidence that the endodomains of other proteins that undergo RIP also have different fates depending on the cellular microenvironment (30).

There have been limited studies of the fate of ACE2 endodomain following ACE2 shedding. ACE2 seems to exhibit different internalization patterns under different stimulation (19, 24, 55, 72, 73). Heurich et al. (19) observed degradation of ACE2 endodomain following the cleavage by ADAM17. Other studies further suggest that during constitutive, low level of ACE2 shedding, mediated by ADAM17, the cytosolic ACE2 fragment is degraded *via* proteasome (19, 24, 55). Moreover, exposure of ACE2-GFP-transfected Neuro-2A cells, a murine neuroblastoma cell line, to high concentrations of Ang II triggered ACE2 internalization and degradation into lysosomes (73). ACE2-internalization triggered by high levels of Ang II is dependent on the presence of AT1R; this study showed that losartan, blocker of AT1R or the absence of AT1R in HEK293T cells failed to facilitate ACE2 internalization (73). ACE2 degradation in lysosomes could be blocked by leupeptin, inhibitor of lysosome and was dependent on the ubiquitination of ACE2 endodomain, triggered by high levels of Ang II (73). Ubiquitination, a major mechanism known to mediate plasma membrane protein internalization involves the addition of ubiquitin moiety to lysine residues (72, 74). E3 ligases that ubiquitinate ACE2 includes MDM2 (murine double minutes 2) in vascular endothelial cells and Skp2 in lung epithelial cells (75, 76). Work by Shen et al. showed that increased levels of MDM2 in lung tissues or pulmonary arterial endothelial cells from patients with idiopathic pulmonary arterial hypertension inversely associated with the levels of ACE2 (75). Furthermore, siRNA specific for MDM2 could increase ACE2 expression while overexpression of MDM2 resulted in significant decrease in ACE2 level in human pulmonary artery endothelial cells (75). Up-regulating Skp2 by bezo-α-pyrene also resulted in reduced cellular ACE2 protein level in 16HBE, human bronchial epithelial cell line, *via* increased ubiquitination of ACE2 (76). To summarize, ubiquitination is a major mechanism involved in regulating cellular ACE2 protein level. In response to increased Ang II, ubiquitination of ACE2 endodomain and interaction of ACE2 with AT1R are critical for the endocytosis and degradation of ACE2 in lysosomes. The interacting AT1R was shown to be recycled and transported back to the membrane (72, 73). ACE2 was shown also to be internalized in response to the binding of the Sp from SAR-CoV-1 and SARS-CoV-2 (19, 24, 55, 77), perhaps another mechanism to reduce surface ACE2 expression. The route of internalization of the ACE2/SARS-CoV-2 complex remains under debate, with reports of clathrin- and caveolae-independent pathways in HEK293T cells, as well as clathrin-dependent and ACE-2 C-terminus-independent internalization in COS7, HepG2 and HEK293T-ACE2 cell lines (77–79). Bayati et al. observed that after engagement with the ACE2 on cell surface, the purified spike glycoprotein and the lentivirus pseudotyped with the Sp of SARS-CoV-2 underwent rapid, clathrin-mediated endocytosis, co-localized with the Rab5-positive early endosomes in HEK293T-ACE2, Vero-SF-ACF and A549 cell lines (79). Inhibitors of clathrin-coated pit formation, such as dynasore and pistop-2 or siRNA against the heavy chain of clathrin blocked the internalization of the SARS-CoV-2 Sp (79). Inoue et al. made similar observations with the Sp of SARS-CoV in COS-7 and HepG2 cell lines and further demonstrated that a low pH condition is required for SARS-CoV to establish an infection and that the endodomain of ACE2 is not required for the internalization of the SARS-CoV-Sp-pseudotyped lentivirus (78). This finding contradicts with what Haga et al. found that the ACE2 cytoplasmic tail is essential for the SARS-CoV Sp induced shedding of ACE2 ectodomain which is required for the endocytosis of SARS-CoV Sp (20). Unfortunately, these two studies did not examine ACE2-shedding or the fate of ACE2 following its internalization. In a seemingly contradictory study of the internalization of SARS-CoV Sp and Sp-pseudotyped lentivirus, Wang et al. found that SARS-CoV bound to ACE2 translocated from cell surface to early endosomes *via* a pH-dependent, clathrin- and caveolae-independent mechanism (77). In HEK293E-ACE2-GFP cells, treatment with ammonium chloride, bafilomycin A1, or chloroquine, resulted in accumulation of ACE2 within perinuclear vacuoles, even after a 14h-incubation. The use of siRNA against clathrin or chlorpromazine, a cationic amphiphilic drug that disrupts clathrin-mediated endocytosis had little inhibitory effects (~20%) on the infectivity of the Sp-pseudotyped lentivirus of VERO cells, suggesting that Sp-ACE2 complex could be internalized *via* a clathrin-independent mechanism. The study further concluded that the 14h-incubation allowed ACE2 to be ‘recycled’ to the cell surface (77). However, the study did not provide strong data to support this conclusion. The ACE2 observed at 14h-post-exposure to Sp or Sp-pseudotyped lentivirus could be newly synthesized ACE2. Blockers of protein-synthesis or protein trafficking should be used to investigate whether ACE2 was recycled back out to the cell surface. Although these studies provide evidence that ACE2 can be internalized *via* different mechanisms, the fate of internalized ACE2 or ACE2 endodomain remains to be sought.

**Figure 2:**
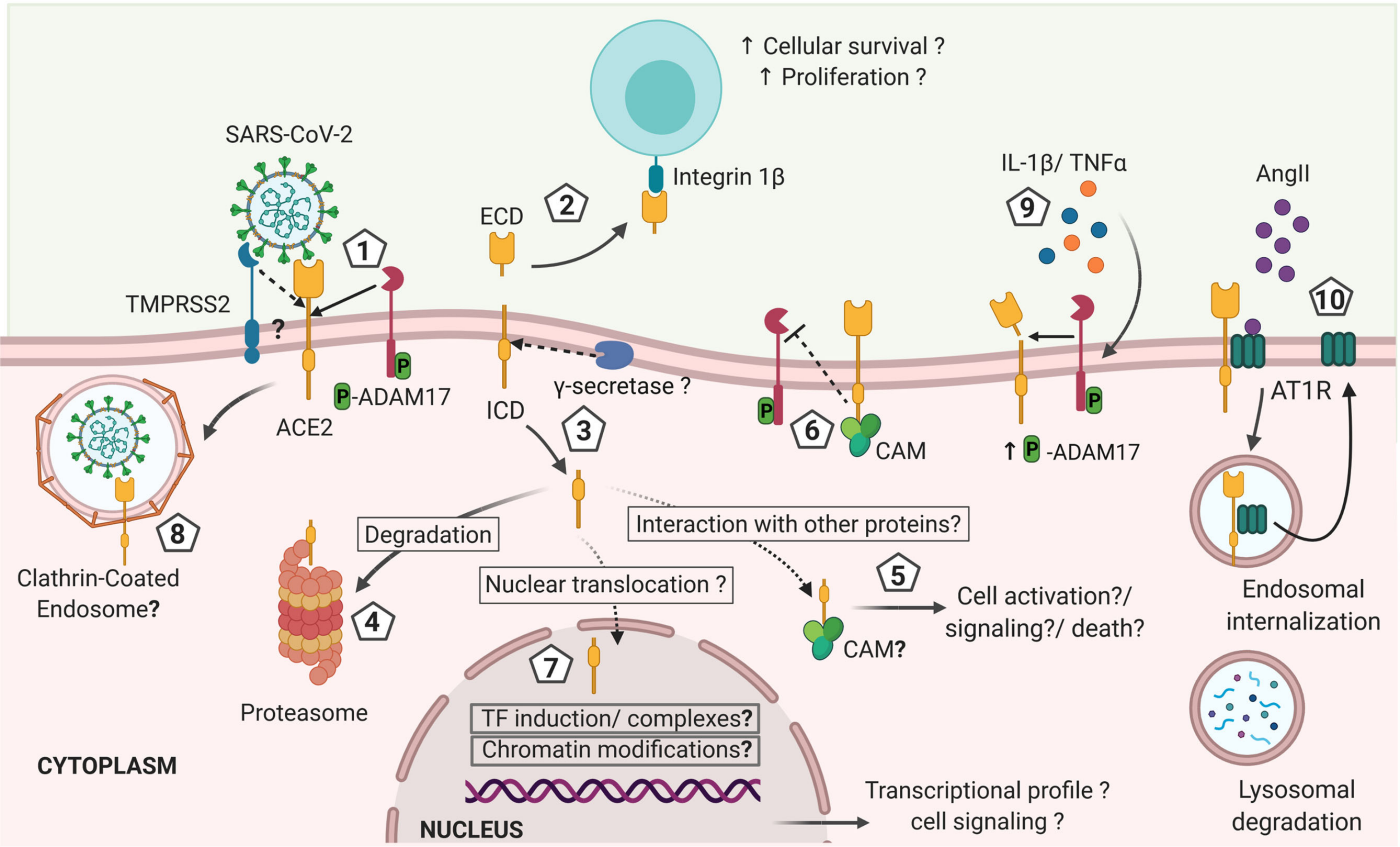
Hypothetical model of ACE2 shedding by ADAM17 and γ-secretase after ACE2 interaction with SARS-CoV-2 and its effects on the immune response against the infection. 1). Initial interaction between SARS-CoV-2 spike protein and the transmembrane cellular receptor ACE2 may trigger proteolytic cleavage of ACE2 mediated by ADAM17, releasing the extracellular domain of this receptor (ectodomain). Other proteases such as TMPRSS2, HAT and Hepsin may also cleave ACE2 at a different catalytic site than ADAM17, thus potentially exhibiting differential outcomes. 2) Soluble ACE2 can interact with other cells through integrins acting like a cell adhesion molecule and triggering signals associated with cell proliferation and survival. 3) The cytoplasmic domain is likely released from the cellular membrane by γ-secretase, as described for other RIP proteins and it is either: 4) degraded at the proteasome, or 5) interacts with cytoplasmic proteins as it has been described for calmodulin (CAM) that can have effects on cellular activation, inflammation and cells death. 6) If CAM interacts with the transmembrane full-length ACE2, the shedding of ACE2 may be abrogated. 7) The intracellularly released ACE2 domain may also be translocated into the nuclei. At the nuclei, the endodomain may induce transcriptional expression of different genes by a still unknown mechanism that can include induction of transcription factors or formation of transcription factors-like complexes or chromatin modifications, influencing the cellular response and fate. In addition, the increased levels of IL-1β and TNFα induced during COVID-19 may increase ADAM17 phosphorylation and activity favoring a sustained shedding of ACE2. Thus, the shedding of ACE2 induced by SARS-CoV-2 infection is likely influencing the outcome of COVID-19 by mechanisms that are still unknown and that remain to be explored in order to design preventive and therapeutic approaches (created with BioRender.com).

Furthermore, whether the non-degraded cytosolic ACE2 endodomain has a physiological function *via* interaction with other cytosolic proteins or translocation to the nuclei remains unknown (**Figure 2**). Nonetheless, a candidate nuclear localization sequence has been predicted in the ACE2 endodomain (^769^RKKKNKA^774^) using the NLStradamus (http://www.moseslab.csb.utoronto.ca/NLStradamus/) (80) program. It remains to be shown whether the ACE2 endodomain has any regulatory function on gene expression (81).

The ACE2 endodomain was shown to interact with Calmodulin (CAM) that mediates important cellular processes, such as metabolism, cell death and immune responses (82, 83). Interestingly, interaction of CAM with the intracellular domain of the full-length ACE2 inhibited ACE2 shedding, suggesting a role for the ACE2 intracellular domain in regulating the cleavage of its ectodomain (82, 83). In support, ACE2 mutant lacking the endodomain could not facilitate the proteolysis of ACE2 ectodomain induced by SARS-CoV Sp (20). Although this finding suggests a role played by the ACE2 endodomain in regulating ADAM17 activity in ACE2 shedding, it could be that deletion of the endodomain affected the structural conformation of the ACE2 ectodomain, not accessible to ADAM17 (20). ACE2 studies were mostly performed *in vitro* with cell lines. Cell types, culture condition and stimuli for ACE2 cleavage or internalization may influence the fate of ACE2 endodomain and should be considered in study design and interpretation of data.

Interestingly, CD147, an alternative receptor for SARS-CoV-2, was also shown to be a target protein of RIP in tumor cell lines (84). CD147 cleavage is mediated by ADAM10 (84). The released CD147 ectodomain was shown to induce the activation of fibroblasts (85). Following CD147 shedding, its endodomain is released into cytosol and translocated to lysosomes to be cleaved again to produce a nuclear localizing subunit, which enhances autophagy function *via* the NF-κB–TRAIL–caspase8–ATG3 axis, favoring tumor cell survival (84). It remains to be explored if SARS-CoV-2 infection triggers RIP of CD147 and ACE2 to modulate cellular activity or function.

## ACE2 and Its Potential Immune-Modulatory Functions

ACE2 may contribute to maintaining immune regulation (86). Overexpression of ACE2 in neurons of a mouse model for induced neurogenic hypertension resulted in reduced arterial pressure, and inflammation, indicated by decreased expression of pro-inflammatory cytokine genes such as *TNFA*, *IL-1β* and *IL-6* in hypothalamus (53). All of these cytokines (TNFα, IL-1β, IL-6) were found in high levels in COVID-19 patients and associated with severe disease outcomes (87–91). In agreement, bone marrow derived macrophages from an ACE2-deficient, atherosclerosis mouse model exhibited a higher baseline level of pro-inflammatory cytokines (e.g., TNFα and IL-6) and hyper-responsiveness to LPS and TNF-α stimulation (92). Moreover, in a murine model of experimentally induced lung injury, administration of a high dose of recombinant ACE2 protected the mice from developing severe acute lung failure associated with reduced pro-inflammatory responses. On the contrary, loss of pulmonary ACE2 expression led to pulmonary malfunction with enhanced vascular permeability and lung edema (93). Similarly, in a mouse model, experimental SARS-CoV infection decreased ACE2 expression in the lung that correlated with lung injury. The observed ACE2 reduction was induced by SARS-CoV Sp (94). In summary, a decrease in cellular ACE2 may reduce the susceptibility of cells to SARS-CoV-2 but leads to greater, undesirable immune activation and more severe tissue damage. In contrast, high abundance of ACE2 on the cell membrane is associated with increased susceptibility to viral particles, but with less damage, due to less inflammation. On the note of inflammation, exogenous soluble ACE2 ectodomain was shown to promote inflammation. ACE2 ectodomain was found to bind to the adhesion molecule, integrin β1, enhancing cell-cell interactions and regulating cell induced signaling, including decreased phosphorylation of the focal adhesion kinase (FAK) (95). Moreover, high concentrations of ACE2 ectodomain (0.1 μg/ml or 1μg/ml) could promote Akt expression and its phosphorylation that have implications in promoting cell activation, proliferation and survival. The ACE2 ectodomain concentration tested is approximately 200-fold higher than the baseline circulating soluble ACE2 concentration (1-2ng/ml serum) (41, 96–98). Although high soluble ACE2 level is probably required to offset the effects of endogenous ACE, which is ~100-200 fold higher than the baseline ACE2 level, these findings are encouraging and should be pursued further to test whether soluble ACE2 could affect immune activation directly at physiological or pathogenic concentrations (95).

Most literature showed that ACE2 modulate immune response ‘indirectly,’ *via* its enzymatic function in cleaving Ang II to generate Ang(1-7), which have anti-inflammatory property (54, 86, 99, 100). As Ang II has pro-inflammatory property, *via* reducing Ang II, ACE2 can promote immune quiescence. In support, treatment of rats with Ang(1-7) reduced inflammatory damage of cardiac tissue, which was associated with decreased NF-κB activity and expression of NF-κB-regulated genes (101). Moreover, treating LPS-activated macrophages with Ang(1-7) resulted in reduced expression of pro-inflammatory cytokine genes, such as *TNFA* and *IL-6*, most likely *via* modulating Src kinase activities (29). However, contrasting findings showed that Ang(1-7) treatment of mice with an established inflammatory condition further aggravated the inflammatory response, resulting in pronounced apoptosis and increased NF-κB activity (102). Some *in vitro* studies showed no effect of Ang (1-9) on NF-κB or Akt expression and phosphorylation (95). In discerning the differences between the two inflammatory animal models (cardiac tissue verses renal tissue) and the macrophage study (29, 95, 101, 102), understanding how Ang(1-7) affects NF-κB activity in these two different tissues may help in resolving this discrepancy.

Moreover, the accumulated Ang II level in the absence or reduction of ACE2 was shown to favor pro-inflammatory states. Exposing alveolar epithelial cells to pro-fibrotic apoptotic inducers while inhibiting ACE2 activity resulted in increased Ang II and decreased Ang(1-7) in cell culture which were associated with increased caspase activation, nuclear fragmentation and JNK phosphorylation (103). In fact, Ang II has been shown to have important pro-inflammatory properties, including inducing the expression of adhesion molecules, recruiting inflammatory cells, and increasing the production of pro-inflammatory cytokines, such as IL-1β, IL-18, IFNγ, TNFα and IL-6 (104). In summary, changes in ACE2 expression or enzymatic activity can modulate Ang II level to affect immune regulation. Although these *in vitro* and murine studies suggest a critical role for ACE2 activities in regulating the pro-inflammatory state, the inherent limitations in murine and *in vitro* models however, prevent the extrapolation of these findings to defining the physiological roles of human ACE2.

## Binding of CoV Sp to ACE2 Induces the Proteolytic Cleavage of ACE2 Potentially Influencing the Severity of Infection

As described above, the initial binding of the SARS-CoV Sp or SARS-CoV-2 Sp with ACE2 is the first step for viral entry. In this regard, current studies are evaluating strategies to reduce transmission or severity of COVID-19 through direct blockade of this interaction (105, 106). Interestingly, although both coronaviruses share only ~79% genetic similarity (8), and 76% homology in their amino acids sequences, both SARS-CoV and SARS-CoV-2 harbor crucial amino acid residues at the receptor binding domain (RBD) of S protein required for binding to ACE2 (9). However, the SARS-CoV-2 RBD binds to ACE2 with a 10-20 fold higher affinity than does the SARS-CoV RBD (22, 107). It is highly likely that additional mechanism(s) are involved in SARS-CoV-2 binding to ACE2, since neutralizing antibodies against non-RBD regions are found in COVID-19 patients, a hypothesis that requires further exploration (108).

Binding of the S protein to ACE2 triggers the shedding of ACE2 ectodomain (20), which explains the reduction in cellular membrane ACE2 observed during SARS-CoV infection (94). Such reduction has also been observed *in vitro* in VERO cells, Caco-2 cells and ACE2-transfected HEK293T cells exposed to recombinant S protein (94). Nonetheless, it is yet to be explored if SARS-CoV-2 S protein also triggers ACE2 shedding (109). A recent study supports this hypothesis. Convalescent COVID-19 patients exhibited elevated ACE2 activity in plasma, compared to matched-healthy donors. The plasma ACE2 activity remains high for a long period of time after recovering and the level and length are positively associated with the disease severity (110). It is not clear how ACE2 shedding is regulated in convalescent COVID-19 patients and how plasma ACE2 level affects the recovery process.

Further supports for the potential impacts of ACE2 shedding in immune activation and disease severity came from HNL-63-CoV- (common cold virus) infected VERO cells (20). HNL-63-CoV infection seems to be acquired during childhood and is usually not associated with severe disease (111).

HNL-63-CoV also engages target cells through binding of its S protein to ACE2. However, SARS-CoV Sp binds to ACE2 with higher efficiency than HNL-63-CoV Sp (111). HNL-63-CoV Sp binding to ACE2 did not trigger ACE2 shedding nor ADAM17-activation (20). Shedding most likely accounted for the observed ACE2 down regulation. Glowacka et al. showed that HNL-63-CoV infection had no effects on the surface level of ACE2 on VERO cells and that recombinant SARS-CoV Sp bound to ACE2 and induced ACE2 shedding with higher efficiency than did the HNL-63-CoV Sp. While ACE2 shedding might be associated with disease severity follow coronavirus infection, it was found to be dispensable for the spread of SARS-CoV and HNL-63-CoV (111). The efficiency of S protein binding to ACE2 may be explained in part by the differences in the amino acid sequences between the S proteins from both viruses (112). The affinity of S protein binding to ACE2 may have influence over the activation of ACE2 cleavage and the outcome of infection.

The differential proteolysis of ACE2 could also be attributed to i) a differential requirement of ADAM17 during infection, since silencing of ADAM17 significantly reduced SARS-CoV infection but not HNL-63-CoV (20), and ii) the differential cellular microenvironment requirements for processing S protein. SARS-CoV infection and the cleavage of its S protein depend on the availability of cathepsin L and low endosomal pH, which is not the case for the HNL-63-CoV infection (113, 114). Other additional mechanisms that were not discussed here can also be involved. Thus, ACE2 cleavage induced by SARS-CoV S protein is most likely a unique mechanism exploited by SARS-CoV that may likely influence the viral pathogenesis of the disease. Whether ACE2 shedding mediated by ADAM17 or TMPRSS2 occurs during SARS-CoV-2 infection and if ACE2 proteolysis is a mechanism contributing to the differential pathogenesis and disease outcome caused by SARS-CoV, SARS-CoV-2 and HNL-63-CoV remains to be explored.

ACE2 cleavage was shown to be essential for efficient SARS-CoV infection (20). However, it may not be the case for SARS-CoV-2 infection or spread. Whether ACE2 proteolysis is required for SARS-CoV-2 infection and/or replication remains debatable. Reduced ACE2 surface expression with increased ACE2-shedding and their relationship with COVID-19 disease severity are yet to be evaluated in SARS-CoV-2 infection.

Although ACE2 cleavage by TMPRSS2 enhances the uptake of SARS-CoV (19), the mechanism involved is still unclear. It was proposed that ACE2 fragments harbors internalization signals that may favor a more efficient uptake of SARS-CoV in a cathepsin L-dependent pathway (19). Even though TMPRSS2 has been shown to compete with ADAM17 in cleaving ACE2, ADAM17-mediated ACE2 cleavage had no effect on the efficiency of viral uptake (19, 33). Work is ongoing in determining the differences in the ACE2 fragments released by TMPRSS2 versus ADAM17.

Furthermore, the establishment of a pro-inflammatory environment as it is observed during SARS-CoV-2 infection may also play a role in triggering ACE2 shedding *via* up regulation of ADAM17 activity. *In vitro* activation of human airway epithelial cells by PMA/Ionomycin or IL-1β and TNFα resulted in increased ADAM17 proteolytic activity and ACE2 shedding (55). The increased plasma levels of both IL-1β and TNFα in COVID-19 patients (115, 116) suggest their potential contribution to increased ADAM17-mediated ACE2 shedding and consequently increased plasma Ang II and heightened renin-angiotensin system, leading to enhanced inflammation and triggering lung failure in COVID-19 patients. However, this hypothesis remains to be tested. An approximated scenario derived from a mouse model of induced acute lung injury showed that SARS-CoV Sp could reduce ACE2 expression and enhance the severity of lung damage by inducing a pro-inflammatory state (94).

Several questions remain unsolved regarding ACE2 proteolysis during CoV infection, especially in SARS-CoV-2, including: 1) Is ACE2 cleavage triggered by SARS-CoV-2 infection? If so, 2) is ACE2 cleavage a RIP event? 3) Are the extracellular and intracellular ACE2 subunits involved in regulation of cellular transcription, signaling and fate? 4) Is the exacerbated immune response and disease severity influenced by ACE2 proteolysis by ADAM17 or TMPRSS2? The answers to these questions could be key for understanding the immunopathogenesis of COVID-19 and for directing therapeutic approaches.

## Conclusions

Although ACE2 shedding is not a novel concept, its regulation during coronavirus infection and the functional roles of the virus-induced generation of ACE2 ectodomain and endodomain remain to be defined. Understanding the functional properties of these domains induced by coronavirus infection will enhance our knowledge in the pathogenesis, potentially mediated by ACE2 shedding and hence, facilitate the design of preventative tools against a severe disease outcome.

Collectively, these supporting evidence suggest that ACE2 is an important regulator of the immune activation, ACE2 shedding, loss of surface ACE2 expression or loss of ACE2 enzymatic activities can result in enhanced immune activation, and pro-inflammatory milieu. The mechanisms underlying these correlative observations are yet to be defined. As ACE2 shares certain features with other RIP proteins, it is highly plausible that the ACE2 intracellular domain has a key regulatory function, responsible for the trans-activation of pro-inflammatory genes in epithelial cells and alveolar macrophages. The contribution of ACE2 shedding to the cytokine storm observed during SARS-CoV-2 requires urgent investigation. It remains to be confirmed whether there is a functional or consequential difference in the ACE2 shedding by ADAM17 versus TMPRSS2. While there are studies on the cleavage of ACE2 by ADAM17, little is known of TMPRSS2-mediated ACE2 shedding, including whether the TMPRSS2-cleaved ACE2 ectodomain enhances the virulence of coronavirus. This knowledge is key to employing inhibition of ACE2 shedding as a therapeutic strategy during early COVID-19 to avoid excessive inflammation and disease severity, caused by lung damages.

## Author Contributions

SG participated in the searching and reviewing of research articles, defining the hypothesis, writing and revising the manuscript, and designing the figures. AS participated in the searching and reviewing of research articles, writing and revising the manuscript. R-CS participated in the searching and reviewing of research articles, defining the hypothesis and writing, reviewing and revising the manuscript. All authors contributed to the article and approved the submitted version.

## Funding

The work is funded primarily by the Public Health Agency of Canada. SMG is supported by the operating grant from Canadian Institute of Health Research to R-CS (CIHR/NIH-154043).

## Conflict of Interest

The authors declare that the research was conducted in the absence of any commercial or financial relationships that could be construed as a potential conflict of interest.
